# Correction: Human-Prosthetic Interaction (HumanIT): A study protocol for a clinical trial evaluating brain neuroplasticity and functional performance after lower limb loss

**DOI:** 10.1371/journal.pone.0318371

**Published:** 2025-01-24

**Authors:** Elke Lathouwers, Bruno Tassignon, Alexandre Maricot, Felipe Gomez, Fanny Delquignies, Claire Cherelle, Ahmed Radwan, Maarten Naeyaert, Hubert Raeymaekers, Peter Van Schuerbeek, Stefan Sunaert, Johan De Mey, Kevin De Pauw

Felipe Gomez, Fanny Delquignies and Claire Cherelle should be included in the author byline. Felipe Gomez, Claire Cherelle, and Fanny Delquignies should be listed as the fourth, fifth and sixth authors respectively. The correct citation is: Lathouwers E, Tassignon B, Maricot A, Gomez F, Delquignies F, Cherelle C, et al. (2024) Human-Prosthetic Interaction (HumanIT): A study protocol for a clinical trial evaluating brain neuroplasticity and functional performance after lower limb loss. PLOS ONE 19(3): e0299869. https://doi.org/10.1371/journal.pone.0299869.

As a result of the correction to the byline, there are errors in the author affiliations, contributions and Competing interests. The correct affiliations are as follows:

Elke Lathouwers^1,2^, Bruno Tassignon^1^, Alexandre Maricot^1^, Felipe Gomez^3^, Fanny Delquignies^3^, Claire Cherelle^3^, Ahmed Radwan^4^, Maarten Naeyaert^5^, Hubert Raeymaekers^5^, Peter Van Schuerbeek^5^, Stefan Sunaert^4,6^, Johan De Mey^5^, Kevin De Pauw^1,2,7^

**1** Human Physiology and Sports Physiotherapy research group, Vrije Universiteit Brussel, Brussels, Belgium, **2** BruBotics, Vrije Universiteit Brussel, Brussels, Belgium, **3** Axiles Bionics, 1130 Brussels, Belgium, **4** KU Leuven, Department of Imaging and pathology, Translational MRI, Leuven, Belgium, **5** Department of Radiology and Magnetic Resonance, UZ Brussel, Belgium, **6** UZ Leuven, Department of Radiology, Leuven, Belgium, **7** Strategic Research Program ‘Exercise and the Brain in Health & Disease: The Added Value of Human-Centered Robotics’, Vrije Universiteit Brussel, Brussels, Belgium.

The correct contributions are:

**Conceptualization:** Elke Lathouwers, Bruno Tassignon, Felipe Gomez, Fanny Delquignies, Claire Cherelle, Kevin De Pauw.

**Funding acquisition:** Kevin De Pauw.

**Methodology:** Elke Lathouwers, Bruno Tassignon, Alexandre Maricot, Ahmed Radwan,

Maarten Naeyaert, Hubert Raeymaekers, Peter Van Schuerbeek, Stefan Sunaert, Johan De

Mey.

**Resources:** Maarten Naeyaert, Hubert Raeymaekers, Peter Van Schuerbeek, Stefan Sunaert,

Johan De Mey, Kevin De Pauw.

**Software:** Ahmed Radwan, Maarten Naeyaert, Hubert Raeymaekers, Peter Van Schuerbeek,

Stefan Sunaert, Johan De Mey.

**Supervision:** Kevin De Pauw.

**Writing–original draft:** Elke Lathouwers.

**Writing–review & editing:** Elke Lathouwers, Bruno Tassignon, Alexandre Maricot, Felipe Gomez, Fanny Delquignies, Claire Cherelle, Ahmed Radwan, Maarten Naeyaert, Hubert Raeymaekers, Peter Van Schuerbeek, Stefan Sunaert, Johan De Mey, Kevin De Pauw.

The correct Competing Interests statement is as follows: Felipe Gomez, Claire Cherelle, and Fanny Delquignies are employees of the prosthetic manufacturer Axiles Bionics. All three authors have been involved in the conceptualisation phase. Fanny Delquignies was also be involved in the data collection phase. None of these three authors were involved in the data analysis phase. Axiles Bionics provided prosthetic feet to conduct this study. This does not alter our adherence to PLOS ONE policies on sharing data and materials. There are no patents, products in development or marketed products associated with this research to declare.
