# Supplementary material for: Human-Prosthetic Interaction (HumanIT): A study protocol for a clinical trial evaluating brain neuroplasticity and functional performance after lower limb loss
Source: PLoS One. 2024 Mar 21;19(3):e0299869. doi: 10.1371/journal.pone.0299869 (PMC10956762; doi:10.1371/journal.pone.0299869)
Supplement: S1 Table — (DOCX) [file pone.0299869.s001.docx]

| **Data category** | **Information** |
| --- | --- |
| Primary registry & trial identifying number | ClinicalTrials.gov: NCT05818410 |
| Date of registration in primary registry | April 17, 2023 |
| Secondary identifying numbers | BUN 1432023000077, EC2023-089 |
| Protocol version | Version 2 – issue date: August 17, 2023 |
| Source(s) of monetary or material support | Vrije Universiteit Brussel  University Hospital Brussels (UZ Brussel)  Axiles Bionics |
| Sponsor | Vrije Universiteit Brussel |
| Contact for public and scientific queries | EL ([Elke.Lathouwers@vub.be](mailto:Elke.Lathouwers@vub.be))  KDP ([Kevin.De.Pauw@vub.be](mailto:Kevin.De.Pauw@vub.be)) |
| Title | Human-Prosthetic Interaction: A study protocol for a clinical trial evaluating brain & technology after lower-limb loss |
| Country of recruitment | Belgium |
| Health condition studied or problem studied | Prosthetic treatment, transtibial amputation, brain neuroplasticity |
| Intervention | Standard of care: SACH® ankle-foot prosthesis  Active comparator: Lunaris® ankle-foot prosthesis |
| Key inclusion and exclusion criteria | Ages eligible for study 25-65 years, sexes available: both; Accepts healthy volunteers: yes |
|  | Inclusion criteria: Adult patient aged 25-65 with a unilateral transtibial amputation & Medicare Functional Classification level ≥ K3 |
|  | Exclusion criteria: Medicare Functional Classification level < K3, metal implants, bilateral amputation, additional upper-limb amputation or diabetes, neurological disorders, excessive stump pains and wounds. |
| Study type | Interventional |
|  | Allocation: alternated, Intervention model: parallel assignment |
| Estimated date of first enrolment | September 2023 |
| Target sample size | 40 individuals with below-knee amputation; 20 able-bodied individuals |
| Recruitment status | Recruiting |
| Primary outcomes | Brain neuroplasticity (Magnetic resonance imaging), Functional performance (distance covered during 6 minute’s walking test |
| Key secondary outcomes | Functional performance (Time to complete test & accuracy), Biomechanical gait parameters (joint angles, velocities and continuous relative phases) and quality of life (questionnaire) |

**S1 Table. Trial registration data.**
