# Supplementary material for: Human-Prosthetic Interaction (HumanIT): A study protocol for a clinical trial evaluating brain neuroplasticity and functional performance after lower limb loss
Source: PLoS One. 2024 Mar 21;19(3):e0299869. doi: 10.1371/journal.pone.0299869 (PMC10956762; doi:10.1371/journal.pone.0299869)
Supplement: S1 Protocol — (PDF) [file pone.0299869.s002.pdf]

CLINICAL INVESTIGATION PLAN

Protocol Title: Human-Prosthetic Interaction: Brain & Technology After Lower-Limb Loss

Coordinating/Principal Investigator: Prof dr. Kevin De Pauw, Prof dr. Guy Nagels, Prof dr. Johan De Mey

I agree:

- to assume responsibility for the proper conduct of this study
- to conduct the study in compliance with this protocol and any future amendments
- not to implement any deviations from or changes to the protocol without prior review and written approval from the Ethics Committee, except where necessary to eliminate an immediate hazard to the subjects, or for administrative aspects of the study (where permitted by all applicable regulatory requirements)
- that I am thoroughly familiar with the appropriate use of the investigational drug, as described in this protocol
- to ensure that all persons assisting me with the study are adequately informed about the investigational drug and their study-related duties and functions as described in the protocol
- that I am aware of and will comply with the current good clinical practice (GCP) guidelines and ethical principles outlined in the Declaration of Helsinki
- to conduct the study in accordance with all applicable laws and regulations

Principal Investigator ______________________________

Kevin De Pauw

Table of contents

1. General **6**
1.1. Introduction 6
1.2. Identification of the clinical investigation plan 6
a) Title of the clinical investigation 6
b) Reference number identifying the specific clinical investigation 6
c) Version of date of the CIP 6
d) Summary of the revision history in the case of amendments 6
e) Version / issue number and reference number 6
f) Abbreviations and acronyms 6
1.3. Sponsor 6
1.4. Principal investigator, coordinating investigator and investigation site(s) 6
a) Name, address, contact details and professional position 6
b) Name and address of the investigation site(s) in which the clinical investigation will be conducted 6
c) Name and address of external organizations involved in the clinical investigation 6
1.5. Overall synopsis of the clinical investigation 6

2. Identification and description of the investigational device **7**
a) Summary description of the investigation device 7
b) Details concerning the manufacturer of the investigational device 7
c) Name or number of the model/type 7
d) Description as to how traceability shall be achieved during and after the clinical investigation 7
e) Intended purpose of the investigational device in the proposed clinical investigation 7
f) The populations and indications for which the investigational device is intended. 7
g) Description of the investigational device 7
h) Summary of the necessary training and experience needed to use the investigational device based on risk assessment 7
i) Description of the specific medical or surgical procedures involved in the use of the investigational device 7
j) References to the IB and IFU 7

3. Justification for the design of the clinical investigation **7**

4. Benefits and risks of the investigational device, clinical procedure, and clinical investigation **7**
a) Anticipated clinical benefits 7
b) Anticipated adverse device effects 7
c) Risks associated with participation in the clinical investigation 7
d) Possible interaction with concomitant medical treatments as considered under the risk analysis 7
e) Steps that will be taken to control or mitigate the risks 7
f) Rationale for benefit-risk ratio 7

5. Objectives and hypotheses of the clinical investigation **7**
a) The purpose of the clinical investigation 7
b) Objectives 7
c) Scientific justification and clinical relevance for effect sizes 7
d) Primary and secondary hypotheses 7

e) Risks and anticipated adverse device effects that are to be assessed 7

6. Design of the clinical investigation 8

6.1. General 8

a) Description of the design type of clinical investigation to be performed 8

b) Description of the measure to be taken to minimize or to avoid bias 8

c) Endpoints 8

d) Methods and timing for assessing, recording and analyzing variables 8

e) Equipment to be used for assessing the clinical investigation variables 8

f) Any procedures for the replacement of subjects 8

g) Investigation site 8

h) Definition of completion of the clinical investigation 8

6.2. Investigation device(s) and comparator(s) 8

a) Description of the exposure to the investigational device 8

b) List of any other medical device or medication to be used 8

c) Number of investigational devices to be used 8

6.3. Subjects 8

a) Inclusion criteria for subject selection 8

b) Exclusion criteria for subject selection 8

c) Criteria and procedures for subject withdrawal or lost to follow-up 8

d) Point of enrolment 8

e) Point of randomization 8

f) Total expected duration of the clinical investigation 8

g) Expected duration of each subject's participation 8

h) Number of subjects required to be included in the clinical investigation 8

i) Estimated time needed to select this number (enrolment period) 8

j) Relationship of investigation population to target population 8

k) Information on vulnerable, pregnant, and breastfeeding population 8

6.4. Procedures 9

a) Description of all clinical investigation-related procedures that subjects undergo during the clinical investigation 9

b) Description of those activities performed by sponsor representative 9

c) Any known or foreseeable factors that can compromise the outcome of the clinical investigation or the interpretation of results 9

d) The methods for addressing these factors in the clinical investigation 9

e) The follow-up period during the clinical investigation 9

f) Address what specific medical care is appropriate to be provided for the subjects after the clinical investigation has been completed 9

g) Address recommended follow-up for the subjects after the clinical investigation has been completed 9

h) Address the final disposition or potential future use of samples obtained from subjects 9

6.5. Monitoring plan 9

7. Statistical design and analysis 9

a) Analysis population 9

b) Descriptive statistics of baseline data 9

c) Analytical procedures including measures of precision such as confidence intervals 9

d) The significance level of the power of primary endpoint(s) 9
e) Sample size calculation and justification 9
f) The rationale for the number of procedures to be performed by a single user as part of the learning curves 9
g) Pass / Fail criteria to be applied to the results of the clinical inversion 9
h) The provision for an interim analysis 9
i) Management of bias 9
j) Management of potential confounding factors 9
k) Description of procedures for multiplicity control and adjustment of error probabilities 9
l) The specification of subgroups for analysis 9
m) Management, justification and documentation of missing data 9
n) Exploratory analysis and sensitivity analysis 9
o) Procedures for reporting any deviation(s) from the original statistical analysis plan 9
p) A strategy for handling the potential imbalance of the number of subjects across investigation sites 9
q) A strategy for pooling data 9

8. Data management 10
a) Methods (e.g. CRF) for data entry and collection 10
b) Procedures used for CRF tracking 10
c) Procedures for verification, validation and securing of electronic clinical data systems 10
d) Procedures to maintain and protect subject privacy 10
e) Methods for database locking at the start of the analysis and storage upon completion of the clinical investigation 10
f) Procedures for data retention 10
g) Specified retention period 10
h) Other aspects of clinical quality assurance 10

9. Amendments to the Clinical Investigation Plan 10

10. Deviations from Clinical Investigation Plan 10
a) Statement specifying that the investigator is not allowed to deviate from the CIP 10
b) Procedures for recording, reporting and analysing CIP deviations 10
c) Notification requirements and time frames 10
d) Corrective and preventive actions and principal investigator disqualification criteria 10

11. Device accountability 10
a) Description of the procedures for the accountability of investigational devices 10
b) Procedures and particular materials and instructions for the safe return of investigational devices 10

12. Statements of compliance 10

13. Informed consent process 10
a) Description of the general process for obtaining informed consent 10
b) Description of the informed consent process in circumstances where the subject is unable to give it 10

14. Adverse events, adverse device effects, and device deficiencies 11
a) Definition of adverse events and adverse device effects 11

b) Definition of device deficiencies 11
c) Definition of serious adverse events 11
d) List of non-reportable adverse events 11
e) Time period in which the principal investigator shall report all adverse events and device deficiencies to the sponsor 11
f) Details of the process for reporting adverse events 11
g) Details of the process for reporting device deficiencies 11
h) List of foreseeable adverse events and anticipated adverse device effects 11
i) Emergency contact details for reporting serious adverse events and serious adverse device effects 11
j) Information regarding the DMC 11

15. Vulnerable population 11

16. Suspension or premature termination of the clinical investigation 11
a) Criteria and arrangements for suspension or premature termination of the whole clinical investigation or of the clinical investigation in one or more investigation sites 11
b) Criteria for access to and breaking the blinding / masking code in the case of suspension or premature termination of the clinical investigation 11
c) Requirements for subject follow-up and continued care 11

17. Publication policy 11

18. Bibliography 11

1. General

1.1. Introduction

The purpose of the Clinical Investigation Plan (CIP) is to provide an overview of good clinical practice for the design, conduct, recording and reporting of clinical investigations carried out in human subjects to assess the clinical effectiveness and safety of the device.

The information contained in this document is the property of the Sponsor/Coordinating Investigator and may not be reproduced, published or disclosed to others without written authorization of the Sponsor/Coordinating Investigator.

This template has been revised to be consistent with ISO 14155:2020 and MEDDEV 2.7/2 revision 2.

1.2. Identification of the clinical investigation plan

a) Title of the clinical investigation

Human-Prosthetic Interaction: Brain & Technology After Lower-Limb Loss

b) Reference number identifying the specific clinical investigation

ClinicTrials.gov Registry Number: Requested

c) Version or date of the CIP

Protocol version and date: Version 1 - 13 January 2023

d) Summary of the revision history in the case of amendments

Version	Date	Comments
1	13-JAN-2023	Initial version

e) Version / issue number and reference number

Version 1 - 13 January 2023.

f) Abbreviations and acronyms

ADL Activities of Daily Living
CIP Clinical Investigation Plan
CIR Clinical Investigation Report
DMC Data Monitoring Committee
H Hypotheses
IB Investigator's Brochure
IFU Instruction For Use
MDR Medical Device Regulation
MRI Magnetic Resonance Imaging
PEQ Prosthetic Evaluation Questionnaire

QUEST Quebec User Evaluation of Satisfaction with assistive Technology
ROs Research Objectives
VUB Vrije Universiteit Brussel

1.3. Sponsor

Vrije Universiteit Brussel, Pleinlaan 2 - 1050 Brussel

1.4. Principal investigator, coordinating investigator and investigation site(s)

a) Name, address, contact details and professional position

Coordinating investigator: Prof dr. Kevin De Pauw, Pleinlaan 2 - 1050 Elsene, kevin.de.pauw@vub.be
Prof dr. Guy Nagels, Pleinlaan 2 -1050 Elsene, Guy.Nagels@vub.be
Prof dr. Johan De Mey, Laarbeeklaan 101, 1090 Jette, Johan.DeMey@uzbrussel.be
Sponsor: Vrije Universiteit Brussel, Pleinlaan 2 - 1050 Brussel
Principal investigator: Prof dr. Kevin De Pauw, Pleinlaan 2 - 1050 Elsene, kevin.de.pauw@vub.be
Study coordinator: Prof dr. Kevin De Pauw, Pleinlaan 2 - 1050 Elsene, kevin.de.pauw@vub.be
Co-investigator: Elke Lathouwers, Generaal Jacqueslaan 271 - 1050 Elsene, Elke.Lathouwers@vub.be
Fanny Delquignies, Raketstraat 64, 1130 Brussel, fanny@axilesbionics.com

b) Name and address of the investigation site(s) in which the clinical investigation will be conducted

UZ Brussel - Laarbeeklaan 101, 1090 Jette
Axiles Bionics - Raketstraat 64, 1130 Brussel

c) Name and address of external organisations involved in the clinical investigation

CHU Brugmann Queen Astrid - Bruynstraat 1, 1120 Neder-over-Heembeek
UZ Leuven campus Pellenberg - Weligerveld 1, 3212 Lubbeek
Ghent University Hospital - Corneel Heymanslaan 10, 9000 Gent
Centre de Traumatologie et de Réadaptation - Arthur Van Gehuchtenplein 4 - 1020 Brussel
Revarte - Eikenstraat 659 - 2650 Edegem

The sponsor will maintain an updated list of investigation sites and investigators. This list will be kept separately from the CIP. The definitive list will be provided with the Clinical Investigation Report (CIR).

1.5. Overall synopsis of the clinical investigation

Official title	Human-Prosthetic Interaction: Brain & Technology After Lower-Limb Loss
Objectives	The main objective of this investigation is to compare rehabilitation with the LUNARIS® and with usual rigid passive feet.
Endpoints	Evaluating functional performance and structural brain adaptions when doing rehabilitation with an articulated ankle-foot prosthesis in comparison with a non-articulation prosthesis.
Study population	Healthy people with lower-limb amputation
Number of subjects	40 lower-limb amputees

	20 able-bodied persons
Inclusion criteria	• Age: 25 - 65 years<br>• Gender: men and women<br>• Level of amputation: unilateral transtibial<br>• Medicare Functional Classification Level: K3-K4
Exclusion criteria	• Having metal implants<br>• Bilateral or additional upper-limb amputation<br>• Diabetic of amputation<br>• Neurological disorders<br>• Excessive stump pains and wounds
Date of first enrollment	September 2023
Duration of clinical investigation	At least 2 years
Follow-up	No follow-up is included in this study

Table 1: Overall synopsis of the clinical investigation

2. Identification and description of the investigational device

a) Summary description of the investigational device

The LUNARIS® is an external ankle-foot prosthetic component with an articulated ankle-joint and internal sensors. It is intended for the replacement of the ankle-foot to compensate for a lower-limb deficiency. It is to be used exclusively for the non-invasive, exo-prosthetic fitting of the lower limb prosthesis, as prescribed by a healthcare professional. It is designed for single-patient use and supports low to moderate impact use. It is developed to fulfil basic daily activities such as standing, walking, stair ascending and descending, slope ascending and descending, sitting, in indoor and outdoor environments.

The LUNARIS® is delivered with its smart charger, the IO® CHARGER. This smart charger is an accessory intended to charge the LUNARIS® and to transfer the data gathered during the day to the Axiles Bionics secured cloud based service for the purpose of predictive maintenance.

b) Details concerning the manufacturer of the investigational device

Axiles Bionics BV
Raketstraat 64, 1130 Brussel
+32 476 37 82 82
pierre@axilesbionics.com
www.axilesbionics.com

c) Name or number of the model/type

LUNARIS®

d) Description as to how traceability shall be achieved during and after the clinical investigation

The study is going to be registered on ClinicalTrials.gov (reference number: requested) and will be open access. The CIP is outlined and the results will be communicated after termination of the study.

e) Intended purpose of the investigational device in the proposed clinical investigation

The LUNARIS® is an external ankle-foot prosthetic component with an articulated ankle-joint and internal sensors. It is intended for the replacement of the ankle-foot to compensate for a lower-limb deficiency. It is to be used exclusively for the non-invasive, exo-prosthetic fitting of the lower limb prosthesis, as prescribed by a healthcare professional. It is designed for single-patient use and supports low to moderate impact use. It is developed to fulfil basic daily activities such as standing, walking, stair ascending and descending, slope ascending and descending, sitting, in indoor and outdoor environments.

f) The populations and indications for which the investigational device is intended.

The LUNARIS® is indicated for use as a component in a lower limb prosthesis for individuals with a missing lower-limb, for example due to trauma, vascular disease, cancer or congenital deficiency, including:

- Unilateral or bilateral amputation or deficiency
- Unilateral or bilateral knee disarticulation
- Unilateral or bilateral transfemoral amputation or deficiency
- Unilateral hip disarticulation

g) Description of the investigational device

The LUNARIS® is a mechanical device which is a component of an external lower limb prosthesis and which is designed to functionally replace, in part or total, a missing ankle-foot complex.

A lower limb prosthesis is commonly composed of multiple components, all assembled and fitted to the amputee by a certified prosthetist[1]. Fig. 1 depicts the different components of a lower-limb prosthesis, consisting of:

- A custom made gel liner (A) - made by the prosthetist - that is wrapped around the residual limb of the amputee (B). The liner is made out of electrically non-conductive materials. The role of the liner is identical to the role of socks in a shoe.
- A custom made hard socket (C) - made by the prosthetist - that is used to fit the residual limb of the amputee (B). The socket is made of solid electrically non-conductive materials, typically manufactured out of fibreglass, carbon fibre infused with acrylic resin, or thermoplastics. Its role is to serve as an interface or attachment to the body of the amputee. Therefore the socket also includes a suspension system to prevent the artificial limb from falling off when the leg is lifted up or moved during gait.
- The shank of the lower limb prosthesis is made by a pylon tube (D), typically manufactured out of steel, aluminium or carbon fibres. This pylon tube is fitted with universal (pyramid or tube) adaptors (E) on both sides to interface the socket on one side, and the ankle-foot component on the other side. Both the pylon tube and adaptors are provided and configured by the prosthetist.
- Lastly, an ankle-foot prosthetic component (F, also referred to as *prosthetic foot*), provided by a legal manufacturer, is attached by the prosthetist to the pylon tube (D) by the means of the universal adaptor (E). The ankle-foot component is typically covered by a foot cover (G). The principle of operation for dorsiflexion/plantar flexion control can be based on various technologies (i.e., electronically driven, mechanical friction/elastic deflection with no fluid or gas operating media, mechanical friction/elastic deflection with fluid or gas operating media).

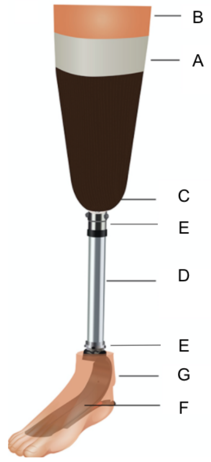

Figure 1: Illustration of a lower limb prosthetic leg with (B) Residual Limb, (A) Gel Liner, (C) Socket, (E) Adaptors, (D) Pylon, (G) Foot Cover and (F) Ankle-Foot Component.

[1] A **Prosthetist** and Orthotist, as defined by The World Health Organisation, is a healthcare professional with overall responsibility of Prosthetics & Orthotics treatment, who can supervise and mentor the practice of other personnel. They are qualified and certified clinicians trained to assess the needs of the user, prescribe treatment, determine the precise technical specifications of prosthesis and Orthosis, take measurements and image of body segments, prepare models of the evaluation, fit devices and evaluate treatment outcome.

It is important to note that **Axiles Bionics only acts as a legal manufacturer of an ankle-foot prosthetic component** (Fig. 1 E-F-G). All other components as described and depicted in Fig. 1 (A-D) are provided and/or made by the prosthetist. The assembly, alignment and tuning to the amputee of the complete lower limb prosthesis is done by the certified prosthetist.

The LUNARIS® is made to fit a normal shoe and connects to the shank (pylon(D) in Fig. 1) through a standard adaptor, which will be used with sockets provided by the healthy subjects. As a result and as explained above, there is no direct contact between the residual limb and the LUNARIS®.

The LUNARIS® is delivered with its smart charger, the IO CHARGER®. This smart charger is an accessory intended to charge the LUNARIS® and to transfer the data gathered during the day to the Axiles Bionics secured cloud based service for the purpose of predictive maintenance.

h) Summary of the necessary training and experience needed to use the investigational device based on risk assessment

The LUNARIS® needs to be fitted and aligned to the lower limb of the subject. Afterwards, basic exercises such as standing, maintaining balance and walking need to be practised so that the subject is able to perform the experimental tasks.

i) Description of the specific medical or surgical procedures involved in the use of the investigational device

Not applicable.

j) References to the IB and IFU

**IB**	IB_HumanIT
**IFU of the LUNARIS®**	MDE002_QA-IFU-D001_FINAL_FR-NL-DE-EN_v1.pdf
**IFU of the IO CHARGER®**	MDE003_QA-IFU-D002_FINAL_FR-NL-DE-EN_v1.pdf

The Investigator Brochure (IB) and the two Instruction For Use (IFU) are attached to this document.

3. Justification for the design of the clinical investigation

A first clinical study, led by the VUB, has been conducted with the LUNARIS® Demonstrator. The complete protocol (B.U.N. 143201526629) has been approved by the medical Ethics Committee of the University Hospital of the VUB.

In this study, forty-two participants completed the protocol assessing performance and functional mobility with their current prosthesis and the LUNARIS® Demonstrator. Subjective measures indicated the added value of this device, while overall task performance and intensity of effort did not differ between the LUNARIS® Demonstrator and the current prosthesis. The familiarisation time only lasted between one and two hours, which was not enough to obtain significant results between the LUNARIS® Demonstrator and the current prosthesis of the user [1].

In addition, a systematic review about therapeutic benefits of lower limb prosthesis was performed in 2021. It could be concluded that no studies investigating the long-term therapeutic benefits of performing daily activities with passive, quasi-passive and active ankle-foot prostheses in people with unilateral lower-limb amputation were found, highlighting a gap in the literature [2].

Lastly, further investigations (B.U.N. 143201526629 - Amendment) unravelling both acute and more prolonged adaptations were conducted to evaluate the LUNARIS® Demonstrator. This study revealed a potential long-term benefit of recovering a gait pattern of able-bodied persons after a few months of familiarisation. We hypothesised that it might be a consequence of the high flexibility of the LUNARIS® Demonstrator [3].

As a result, this study aims to demonstrate that doing the rehabilitation with the LUNARIS® may allow the user to recover a gait pattern closer to the one of able-bodied person and that brain plasticity will be less impacted when walking with the LUNARIS® than with another rigid prosthetic device. Studying the effect of the LUNARIS® during rehabilitation could be a great opportunity to investigate the rehabilitation with an articulated ankle prosthesis and the long term benefits of the LUNARIS®.

4. Benefits and risks of the investigational device, clinical procedure, and clinical investigation

a) Anticipated clinical benefits

The LUNARIS® aims to restore basic functional mobility during daily activities in healthy people with a lower-limb amputation and improve their quality of life. This study aims to demonstrate that doing the rehabilitation with the LUNARIS® may allow the user to recover a gait pattern closer to the one of able-bodied person and that brain plasticity will be less impacted when walking with the LUNARIS® than with another rigid prosthetic device. To the best of the authors' knowledge, currently no studies investigated the long-term therapeutic benefits of prosthetic feet [2]. Studying the effect of the LUNARIS® during rehabilitation could be a great opportunity to fill this gap.

b) Anticipated adverse device effects

There are no disadvantages or risks associated with this research. Moreover, previous experiments with the Talaris Demonstrator (the LUNARIS® prototype, registered on B.U.N. 143201526629) have shown that there is a high willingness and motivation from the participants to participate in the experiments. In addition, no side or adverse effects were reported.

c) Risks associated with participation in the clinical investigation

The LUNARIS® is a CE-marked medical device. Therefore, risk analysis has been performed according to ISO 14971 to ensure that risks associated with the device are identified, evaluated and controlled.

The risk analysis shows that any residual risks which may be associated with the device are acceptable, when weighed against the benefits of the intended use to the lower-limb amputee, and compatible with a high level of protection of health and safety.

d) Possible interaction with concomitant medical treatments as considered under the risk analysis

Not applicable.

e) Steps that will be taken to control or mitigate the risks

We ensure that any event as referred to in Article 80(2) of the Medical Device Regulation will be fully recorded by the investigators, manufacturer or other parties, and immediately notified by the sponsor to all competent authorities (including FAMHP) by using the European form in a timely manner.

f) Rationale for benefit-risk ratio

The overall residual risk associated with the device is acceptable in relation to the benefits of the intended use of the device. The users may expect more benefits than risks in using the device, therefore, the device does not pose a concern to human health for the intended use.

The Risk Management File is attached to this document.

5. Objectives and hypotheses of the clinical investigation

a) The purpose of the clinical investigation

This clinical investigation aims to study the rehabilitation with the LUNARIS® in comparison to the rehabilitation with usual rigid passive feet.

b) Objectives

The different research objectives (ROs) are:

RO.1.1 The primary aim is to investigate the effect of walking performance with a new passive ankle device (LUNARIS®) compared to the current prosthetic ankle device in people with a unilateral lower limb amputation.
RO.1.2 A second aim is to examine the effect of the different prostheses on postural stability in people with a unilateral lower limb amputation.
RO.1.3 A third aim is to determine if the type of prosthesis changes the movement pattern during walking in an individual with a unilateral lower limb amputation.
RO.2 The secondary aim is to examine changes in whole-brain networks related to the type of prosthesis in people with a unilateral lower limb amputation by means of MRI and diffusion tensor imaging.

c) Scientific justification and clinical relevance for effect sizes

The primary endpoint of the clinical trial is the distance covered during the 6-minutes' walk test. The sample size was calculated based on a study comparing passive and quasi-passive prosthetic ankles which found an effect size Cohens' f = 0.318 on distance covered during the 6-minutes' walk test [4]. A total of 40 participants with a lower limb amputation is required and will be allocated to one of the two prosthetic groups. These individuals will be stratified by age (2 strata: < 45 years, ≥ 45 years, block size = 4) to control for age-related cognitive deterioration. A matched control-group of 20 able-bodied individuals will be included to enable comparison. The required number of 40 participants with amputation is based on a sample size calculation for an analysis of variance using G*Power (version 3.1.9.4) envisaging 10% drop-out. Values used for this calculation were medium expected effect size f = 0.318, power = 0.80, alfa = 0.05, the number of groups = 3 and the number of measurements set at 2 with a correlation between measurements of 0.75.

d) Primary and secondary hypotheses

The different hypotheses (H) are:

H1 We hypothesise that people walking with the passive prosthesis (LUNARIS®) will be able to cover significantly more distance (i.e., > 35m) during 6-min walking compared to people walking with their current prosthetic device [4][5]. Secondly, we expect that wearing the LUNARIS® will improve postural stability and improve movement patterns after 12 weeks compared to walking with the current ankle prosthesis [6][7][8]. Thirdly, we noticed based on anecdotal evidence that people walking with the LUNARIS® are able to descend stairs more naturally, like able-bodied people. We hypothesise that doing the rehabilitation with the LUNARIS® will help people descending stairs without the need of rolling over the edge of a step, and as a consequence, will reduce the number of falls.
H2 Based on my literature review on brain neuroplasticity after a lower limb amputation, we hypothesise to discover differences in structural whole-brain networks between participants with the LUNARIS® and current prosthesis after 12 weeks. We assume that these differences in structural networks will be less pronounced among people wearing the LUNARIS® compared to people wearing the current

prostheses [9]. Furthermore, we expect that these disparities in brain structural connectivity will translate into a change in performance on the physical tests.

e) Risks and anticipated adverse device effects that are to be assessed

To date, no device deficiencies have been reported with the LUNARIS® . We are not yet able to give a concise summary of device deficiencies and thus not able to report any deficiency that might have led to a serious adverse event if appropriate action had not been taken, intervention had not occurred, or circumstances had been less fortunate.

The Risk Management File is attached to this document.

6. Design of the clinical investigation

6.1. General

a) Description of the design type of clinical investigation to be performed

This cohort study includes three groups.

- 20 lower-limb amputees who are going to do their rehabilitation and perform daily activities with a new articulated ankle foot prosthesis: the LUNARIS®.
- 20 lower-limb amputees who are going to do their rehabilitation and perform daily activities with a conventional prosthetic ankle: the SACH® followed by the most suited prosthesis for them.
- 20 able-bodied subjects which will be used as a control group.

b) Description of the measure to be taken to minimise or to avoid bias

All appointments for the MRI will take place at UZ Brussel to avoid any bias due to a different MRI scanner. All appointments for functional performance testing will take place at Axiles Bionics to avoid any bias due to different slopes or staircases between subjects and between sessions.

c) Endpoints

Firstly, we will investigate the beneficial effect of an articulated prosthetic ankle (LUNARIS®) compared to an usual rigid passive foot (SACH®) on functional physical performance by means of a clinical investigation. Secondly, this investigation will examine the differences in brain neuroplasticity and movement patterns according to the type of prosthesis.

d) Methods and timing for assessing, recording and analysing variables

Population & sample size:

Adults aged 25-65 with a transtibial amputation will be recruited through contacting rehabilitation centres, and orthopaedic departments of hospitals within the network of BruBotics (BruBotics.eu) and Axiles Bionics (Axiles Bionics | Next Generation Bionics). Adults with a Medicare Functional Classification level lower than K3, metal implants, bilateral amputation, additional upper-limb amputation or diabetes will be excluded as well as participants with neurological disorders or with excessive stump pains and wounds. All participants will be asked to provide their written consent after being written and verbally informed regarding the study protocol. The study will be executed in compliance with the Declaration of Helsinki [10] and Ethical approval will be sought from the medical ethics commission of VUB and UZ Brussel.

Protocol:

Figure 2 provides a visualisation of the investigation workflow.

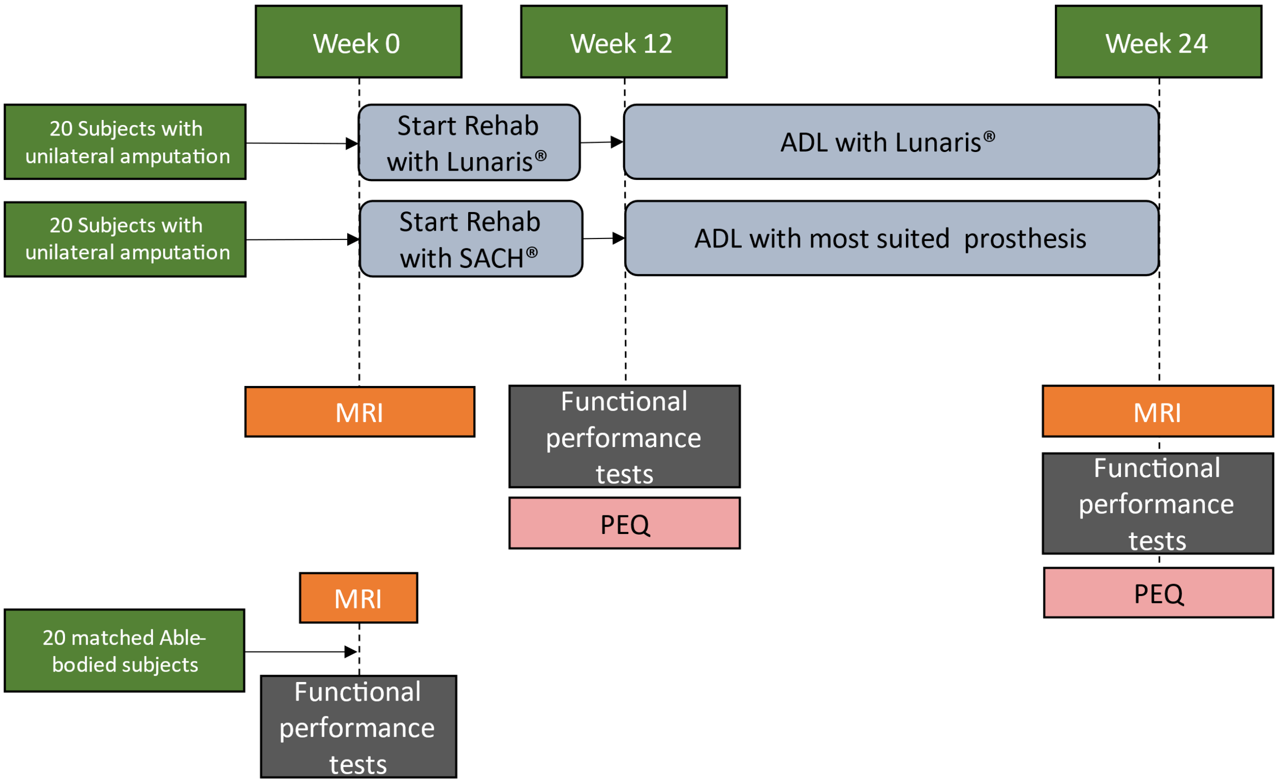

Figure 2: Overview clinical trial. MRI: Magnetic Resonance Imaging, PEQ: Prosthetic Evaluation Questionnaire, ADL: Activities of Daily Living

The clinical investigation will comprise 4 test days for participants with a lower limb amputation and 2 days for the control group of able-bodied individuals.

The able-bodied individuals will undergo an MRI-scan at the University hospital Brussels (dpt. Radiology and Magnetic Resonance) and then perform functional performance tests.

Participants with a lower limb amputation will start the clinical investigation upon the start of their rehabilitation. At week 0, when initiating the rehabilitation, participants will undergo a baseline MRI-scan at the University hospital Brussels (dpt. Radiology and Magnetic Resonance). Then, they will be allocated to the one of the treatment arms (LUNARIS® or the SACH® foot) and will conduct their rehabilitation to learn walking with a prosthesis. At the end of the rehabilitation, after 12 weeks, participants will perform baseline functional performance tests, fill out the prosthetic evaluation questionnaire (PEQ) measuring quality of life [11] and fill out the Mini-Mental State Examination to account for cognitive impairments and onset of Alzheimer's Disease [12]. Between weeks 12 and 24 of the clinical investigation (i.e. intervention period), participants will perform their daily activities with the allocated prosthesis. During this 12-week period, trying out new prosthetic devices will be allowed within the group of individuals wearing the SACH® foot as this is considered the usual care. At the end of this period (after week 24), post-test assessment will take place and participants will undergo the same MRI and functional performance tests as during the baseline assessments to evaluate the changes that occurred. Additionally, the participant will be asked to fill out the Quebec User Evaluation of Satisfaction with assistive Technology (QUEST 2.0) to assess prosthetic satisfaction [25-27]. The 12-week intervention period is chosen based on a study examining the effect of 12 weeks balance training in healthy and older adults on neuroplasticity and on the accommodation time to walking with a new prosthesis [13][14][15].

e) Equipment to be used for assessing the clinical investigation variables

Functional performance testing

Functional balance will be examined. Table 2 lists the different tests. A 6-min walking test, dual-task L-test, L-test, slope walking test, and stairs climbing and descending test will indicate functional balance and dual-task performance. Rating of perceived exertion [16], level of fatigue and comfort [17] and NASA-Task Load index (NASA-TLX) assessing perceived workload [18] will be assessed after each task.

Performance tests	Outcome	Materials
Slope walking test [19]	Time to complete test	Stopwatch
	Biomechanical gait parameters	Inertial measurements units
Stair climbing and descending test [20]	Time to complete test	Stopwatch
	Biomechanical gait parameters	Inertial measurements units
Dual-task L-test [21]	Time to complete test	Stopwatch
	Accuracy of subtractions	Audio-recorder
L-test [21]	Time to complete test	Stopwatch
6-min walking test [5]	Distance covered in 6 minutes	Measuring wheel
	Biomechanical gait parameters	Inertial measurements units

Table 2: Physical performance tests

The primary endpoint is the distance covered during the 6-min walking test. Any difference found between the different prostheses on the distance travelled will be considered clinically relevant from a difference of 35 metres [5]. Secondary endpoints will comprise performance on the L-test, dual-L-test, slope walking test and stairs climbing and descending test, and the evaluation of the gait pattern.

Initial measurements units (Awinda, Xsens Technologies B.V., Enschede, The Netherlands) will be capturing 3D-accelerations and angular velocities during the 6-min walking test, the slope walking test and the stairs climbing and descending test to evaluate the participants' gait pattern [22]. The data of the inertial measurement units will be used to evaluate coordination patterns between joints or segments based on continuous relative phase plots. We opt to evaluate this, as high variability in the gait pattern increases the risk of falling. Biomechanical data will be analysed and purified using Matlab (The MathWorks Inc., Massachusetts, United States).

Structural brain adaptions using MRI

Structural whole-brain networks changes will be researched by applying different MRI techniques during pre, mid, and post-test assessment. MRI-assessment is part of the clinical investigation of WP1. An MRI scan will be conducted to gather an anatomical scan comprising different sequences on a 3-Tesla MRI-unit. A 30-minute protocol will be followed, compiled out of scout images, 3D T1-weighted spin-echo images, diffusion-weighted imaging HARDI and an optional 3D MAGIC. These measurements will consequently be used to determine whole-brain networks based on the cortical and subcortical regions of interest [23][24]. Regions of interest will include the primary motor cortex, primary somatosensory cortex, premotor cortexes, supplementary motor area, corpus callosum, basal ganglia, thalamus and cerebellum as they are responsible for motor control and balance.

f) Any procedures for the replacement of subjects

Not applicable.

g) Investigation site

UZ Brussel - Laarbeeklaan 101, 1090 Jette

Axiles Bionics - Raketstraat 64, 1130 Brussel

h) Definition of completion of the clinical investigation

The clinical investigation will be considered as terminated when the proposed sample size is tested and is defined as the last visit of the last subject.

6.2. Investigation device(s) and comparator(s)

a) Description of the exposure to the investigational device

20 subjects are asked to do their rehabilitation process with the LUNARIS® and then to perform their daily activities with the LUNARIS® during a period of 6 months in total. They are also asked to perform the above mentioned activities with the LUNARIS® during 2 experimental sessions.

b) List of any other medical device or medication to be used

20 subjects are asked to do their rehabilitation process with an usual rigid passive foot (SACH®) and then to perform their daily activities with the most suited prosthesis for them during a period of 6 months in total. They are also asked to perform the above mentioned activities with the most suited prosthesis for them during 2 experimental sessions.

c) Number of investigational devices to be used

The LUNARIS®, already extensively described in this document and in the investigator brochure, is the only investigational device that will be used.

6.3. Subjects

a) Inclusion criteria for subject selection

- Age: 25 - 65 years
- Gender: men and women
- Level of amputation: unilateral transtibial
- Medicare Functional Classification Level: K3-K4

b) Exclusion criteria for subject selection

- Having metal implants
- Bilateral or additional upper-limb amputation
- Diabetic cause of amputation
- Neurological disorders
- Excessive stump pains and wounds

c) Criteria and procedures for subject withdrawal or lost to follow-up

Participants can withdraw at any time without reason from the experiment. In case of subjects drop-out is not met, additional subjects will be recruited via the same rehabilitation centres and orthopaedics departments of hospitals until the sample size is obtained (40 lower-limb amputees and 20 able-bodied subjects).

d) Point of enrolment

Participants will be recruited via rehabilitation centres and orthopaedics departments of hospitals such as CHU Brugmann Queen Astrid, UZ Leuven campus Pellenberg, Ghent University Hospital, Revarte and Centre de Traumatologie et de Réadaptation. All participants will be providing written and oral information about the experimental protocol, potential risks and benefits before giving informed consent to participate in the study.

e) Point of randomization

Non-randomized.

f) Total expected duration of the clinical investigation

We expect to start the clinical investigation in September 2023. It will last at least for 2 years.

g) Expected duration of each subject's participation

Each subject is asked to schedule four appointments. The total duration of the clinical investigation for each subject is 24 weeks.

h) Number of subjects required to be included in the clinical investigation

We want to include 40 lower-limb amputees and 20 able-bodied subjects. Several rehabilitation centres and orthopaedics departments of hospitals were already contacted and agreed to participate in this study by providing us lower-limb amputees.

i) Estimated time needed to select this number (enrolment period)

Enrolment will be performed before and during the experiment. We expect that it will take less than 2 years to gather enough subjects for this clinical investigation.

j) Relationship of investigation population to target population

The investigation population could be the future target population.

k) Information on vulnerable, pregnant, and breastfeeding population

Not applicable.

6.4. Procedures

a) Description of all clinical investigation-related procedures that subjects undergo during the clinical investigation

Lower-limb amputees:

There are three measurement moments for lower-limb amputees which will be divided into four days.

Day 1: the subject will be expected at the radiology department at UZ Brussel. Here, a non-invasive brain scan will be taken under the supervision of Prof. Dr. Johan De Mey (department head of Radiology). This measurement moment will take approximately 60 minutes.

After 12 weeks: the subject will be expected at Axiles Bionics for a series of non-invasive tests. This measurement moment will take approximately 120 minutes.

After 24 weeks: this measurement moment will be divided into two days. The subject will be expected on day 1 at the radiology department at UZ Brussel. Here, the same brain scan will be taken as described in measurement moment 1. This measurement moment will take about 60 minutes. On day 2, the subject will be expected at Axiles Bionics for a series of non-invasive tests as described in measurement moment 2. This measurement moment will take about 120 minutes.

Able-bodied subjects:

There is only one measurement moment for able-bodied subjects which will be divided into two days.

Day 1: the subject will be expected at the radiology department at UZ Brussel. Here, a non-invasive brain scan will be taken under the supervision of Prof. Dr. Johan De Mey (department head of Radiology). This measurement moment will take approximately 60 minutes.

Day 2: the subject will be expected at Axiles Bionics for a series of non-invasive tests. This measurement moment will take approximately 60 minutes.

b) Description of those activities performed by sponsor representative

The sponsor is responsible for the execution of the experimental tasks, data gathering and analyses.

c) Any known or foreseeable factors that can compromise the outcome of the clinical investigation or the interpretation of results

There are no known or foreseeable factors that can comprise the outcome. Subjects are asked to behave as normal as possible before, during and after the experiment concerning the amount of physical activity and medication intake.

d) The methods for addressing these factors in the clinical investigation

Specific inclusion and exclusion criteria are applied before enrolment to counteract as possible factors that can compromise the outcome.

e) The follow-up period during the clinical investigation

The principal investigator and co–investigators are available during the entire duration of the experiment of each subject. Follow-up is provided for each subject during their participation via direct appointments or via other channels such as email or telephone calls.

f) Address what specific medical care is appropriate to be provided for the subjects after the clinical investigation has been completed

There is no follow-up plan considered since subjects are doing the rehabilitation process as usual and perform their daily activities with the investigational device or with the most suited prosthesis for them. Subjects can contact our department after and during the experiment for any further questions.

g) Address recommended follow-up for the subjects after the clinical investigation has been completed

There is no follow-up plan considered since subjects are doing the rehabilitation process as usual and perform their daily activities with the investigational device or with the most suited prosthesis for them. Subjects can contact our department after and during the experiment for any further questions.

h) Address the final disposition or potential future use of samples obtained from subjects

All anonymized data will be stored for up to 10 years as recommended by the VUB.

6.5. Monitoring plan

The investigators have a certificate of "Good Clinical Practice".

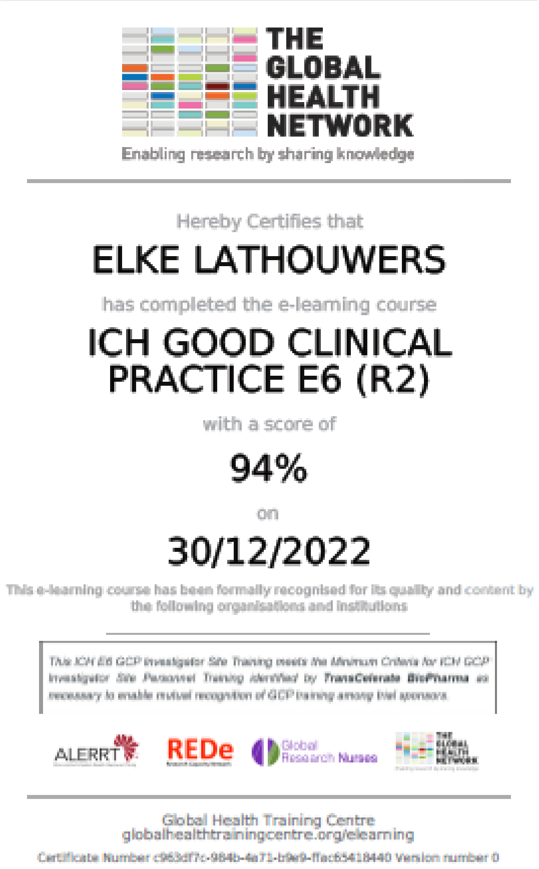
THE GLOBAL HEALTH NETWORK
Enabling research by sharing knowledge

Hereby Certifies that

ELKE LATHOUWERS

has completed the e-learning course

ICH GOOD CLINICAL PRACTICE E6 (R2)

with a score of

94%

on

30/12/2022

This e-learning course has been formally recognised for its quality and content by the following organisations and institutions

This ICH E6 GCP Investigator Site Training meets the Minimum Criteria for ICH GCP Investigator Site Personnel Training identified by **TransCelerate BioPharma** as necessary to enable mutual recognition of GCP training among trial sponsors.

ALERRT REDe Global Research Nurses THE GLOBAL HEALTH NETWORK

Global Health Training Centre
globalhealthtrainingcentre.org/elearning
Certificate Number c963df7c-984b-4a71-b9e9-ffac65418440 Version number 0

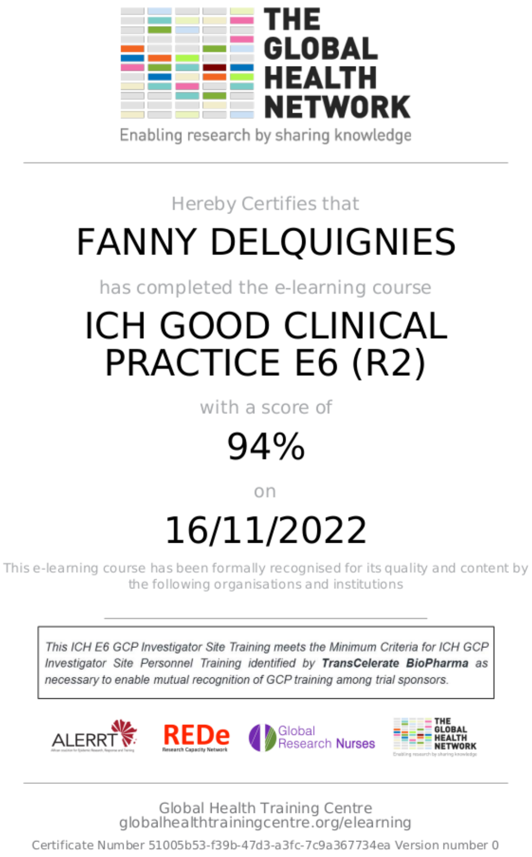
THE GLOBAL HEALTH NETWORK
Enabling research by sharing knowledge

Hereby Certifies that

FANNY DELQUIGNIES

has completed the e-learning course

ICH GOOD CLINICAL PRACTICE E6 (R2)

with a score of

94%

on

16/11/2022

This e-learning course has been formally recognised for its quality and content by the following organisations and institutions

*This ICH E6 GCP Investigator Site Training meets the Minimum Criteria for ICH GCP Investigator Site Personnel Training identified by **TransCelerate BioPharma** as necessary to enable mutual recognition of GCP training among trial sponsors.*

ALERRT REDe Research Capacity Network Global Research Nurses THE GLOBAL HEALTH NETWORK

Global Health Training Centre
globalhealthtrainingcentre.org/elearning
Certificate Number 51005b53-f39b-47d3-a3fc-7c9a367734ea Version number 0

7. Statistical design and analysis

a) Analysis population

The entire population enrolled in the study will be included in the analysis.

b) Descriptive statistics of baseline data

Functional performance testing

A mixed model will be constructed enabling within and between group (i.e., type of prosthesis) comparison of balance performance and accounting for baseline covariates. If residuals are not normally distributed, required adaptations to the data or model will be performed. To correct for multiple comparisons, suited corrections will be applied. The factors age, time since amputation, side of amputation, level of amputation, time using prosthesis, previously used prosthesis will be accounted for. Statistical significance level will be set at 5%.

Structural brain adaptations

Differences in connectivity between pre-test and post-test assessment, both within and between groups (i.e. type of prosthesis), will be analysed by means of structural equation modelling. This analysis allows to investigate the causal relationships between brain structure and performance indicators while controlling for confounding factors. The factors time since amputation, side of amputation, amputation level, time using prosthesis, previously used prosthesis, age, sex, Mini-Mental State Examination score, and level of education will be accounted for. Statistical significance level will be set at 5%.

c) Analytical procedures including measures of precision such as confidence intervals

Not applicable.

d) The significance level of the power of primary endpoint(s)

Statistical significance level will be set at 5%.

e) Sample size calculation and justification

The primary endpoint of the clinical trial is the distance covered during the 6-minutes' walk test. The sample size was calculated based on a study comparing passive and quasi-passive prosthetic ankles which found an effect size Cohens' f = 0.318 on distance covered during the 6-minutes' walk test [4]. A total of 40 participants with a lower limb amputation is required and will be allocated to one of the two prosthetic groups. These individuals will be stratified by age (2 strata: < 45 years, ≥ 45 years, block size = 4) to control for age-related cognitive deterioration. A matched control-group of 20 able-bodied individuals will be included to enable comparison. The required number of 40 participants with amputation is based on a sample size calculation for an analysis of variance using G*Power (version 3.1.9.4) envisaging 10% drop-out. Values used for this calculation were medium expected effect size f = 0.318, power = 0.80, alfa = 0.05, the number of groups = 3 and the number of measurements set at 2 with a correlation between measurements of 0.75.

f) The rationale for the number of procedures to be performed by a single user as part of the learning curves

A baseline experiment with a conventional prosthetic ankle used in rehabilitation is required to observe any similarities or differences in outcomes with the LUNARIS®. Moreover, a baseline experiment with able-bodied subjects is required to observe any similarities or differences in outcomes with the LUNARIS®. Therefore, each lower-limb amputee is asked to schedule four appointments and each able-bodied subject is asked to schedule two appointments to run through the whole experiment.

g) Pass / Fail criteria to be applied to the results of the clinical investigation

Not applicable.

h) The provision for an interim analysis

Not applicable.

i) Management of bias

All appointments for the MRI will take place at UZ Brussel to avoid any bias due to a different MRI scanner. All appointments for functional performance testing will take place at Axiles Bionics to avoid any bias due to different slopes or staircases between subjects and between sessions.

j) Management of potential confounding factors

Not applicable.

k) Description of procedures for multiplicity control and adjustment of error probabilities

The principal investigator and co-investigators will analyse the data independently to identify any error probabilities.

l) The specification of subgroups for analysis

Not applicable.

m) Management, justification and documentation of missing data

Drop-outs and unused data will not be included in the analyses. We ensure that any unused or spurious data (including drop-outs) will be fully recorded by the investigators, manufacturer or other parties, and immediately notified to sponsor to all competent authorities (including FAMHP) by using the European form in a timely manner.

n) Exploratory analysis and sensitivity analysis

Not applicable.

o) Procedures for reporting any deviation(s) from the original statistical analysis plan

We ensure that any deviation will be fully recorded by the investigators, manufacturer or other parties, and immediately notified to sponsor to all competent authorities (including FAMHP) by using the European form in a timely manner.

p) A strategy for handling the potential imbalance of the number of subjects across investigation sites

Not applicable since each subject of this clinical investigation will perform the different experiments in the two same investigation sites.

q) A strategy for pooling data

All data will be entered in excel and physical copies will be scanned as a pdf file. During the investigation, data will be saved on the VUB share point, which has a built-in back-up. After the investigation, data will be anonymized where possible and stored in the VUB university archive.

As personal and sensitive data of human participants will be collected, pseudonymisation of the data will take place as soon as possible upon data acquisition. The file which establishes the link between the pseudonymised identities and the identification of the participant, will be password- protected.

All raw data will be stored in the online system of the Ethical Committee of the UZ Brussel (i.e., Redcap). Raw and cleaned data will also be stored and encrypted on the research's group network attached storage (NAS) under supervision of Prof. Kevin De Pauw. The NAS is system-encrypted, back-upped, has up-to-date antivirus and is only accessible by authorised personnel. Passwords will be required to access the NAS, in compliance with the GDPR regulations. All manual files and written data will be stored in locked filing cabinets. All anonymized data will be stored for up to 10 years as recommended by the VUB.

Option to access the database will require a data use agreement. The stored data will remain unchanged and be identified by the researcher ORCID and article DOI. Transfer of data will only be done if essential.

8. Data management

a) Methods (e.g. CRF) for data entry and collection

All data will be entered in excel and physical copies will be scanned as a pdf file. During the investigation, data will be saved on the VUB share point, which has a built-in back-up. After the investigation, data will be anonymized where possible and stored in the VUB university archive.

Option to access the database will require a data use agreement. The stored data will remain unchanged and be identified by the researcher ORCID and article DOI. Transfer of data will only be done if essential.

b) Procedures used for CRF tracking

The pre-processing (basic filters and artefact removal) steps will be performed by the co-investigators to ensure data quality.

c) Procedures for verification, validation and securing of electronic clinical data systems

The non-invasive brain MRI-scan will be taken under the supervision of Prof. Dr. Johan De Mey, who is the department head of Radiology.

d) Procedures to maintain and protect subject privacy

As personal information is branded by the EU as sensitive, data will be anonymized as soon as possible. Participants will receive a code corresponding to their inclusion order, and the file that relates these codes to their personal information will be encrypted. During the course of the study, data will only be stored with personal access, on a personal cloud server. After the study completion, all data will be shared with the research lab through a network attached storage.

As personal and sensitive data of human participants will be collected, pseudonymisation of the data will take place as soon as possible upon data acquisition. The file which establishes the link between the pseudonymised identities and the identification of the participant, will be password- protected.

The data protection officer of the VUB (dpo@vub.be) will be notified of our study. Data in the present study will be managed confidentially, conforming to the Algemene Verordening Gegevensbescherming (GDPR) of 27 April 2016.

Participating in this study means that the participant agrees that the investigators gather data of the participant that will be used to conduct the investigation and eventually will result in the publications in scientific journals and presentations at conferences. The participant will always have the right to ask the investigators which data he/she has gathered on them and for what this data is used in light of the investigation. The participant retains the right to look into this data and to ask for corrections on the occasion this data would contain errors. The participant also retains the right to stop his/her participation in the study at any moment.

e) Methods for database locking at the start of the analysis and storage upon completion of the clinical investigation

All data will be entered in excel and physical copies will be scanned as a pdf file. During the investigation, data will be saved on the VUB share point, which has a built-in back-up. After the investigation, data will be anonymized where possible and stored in the VUB university archive.

Option to access the database will require a data use agreement. The stored data will remain unchanged and be identified by the researcher ORCID and article DOI. Transfer of data will only be done if essential.

f) Procedures for data retention

All raw data will be stored in the online system of the Ethical Committee of the UZ Brussel (i.e., Redcap). Raw and cleaned data will also be stored and encrypted on the research's group network attached storage (NAS) under supervision of Prof. Kevin De Pauw. The NAS is system-encrypted, back-upped, has up-to-date antivirus and is only accessible by authorised personnel. Passwords will be required to access the NAS, in compliance with the GDPR regulations. All manual files and written data will be stored in locked filing cabinets. All anonymized data will be stored for up to 10 years as recommended by the VUB.

g) Specified retention period

All anonymized data will be stored for up to 10 years as recommended by the VUB.

h) Other aspects of clinical quality assurance

In order to control the quality of the present study, the medical file of the participants can be consulted. If so, this will be done by qualified personnel who are bound to professional secrecy, e.g. representatives of the ethical committee or an external auditing bureau. This can only take place under strict conditions, under the responsibility of the investigator and under his supervision. The encrypted data can also be passed through the Belgium government or another regulatory authority or the ethical committee.

9. Amendments to the Clinical Investigation Plan

The study will be submitted to the commission of medical ethics at the University Hospital of the Vrije Universiteit Brussel. Moreover, it will also be registered in the Clinicaltrials.gov database. Once the clinical investigation is approved, they shall be notified to all proposed changes to the approved clinical investigation that have a substantial impact on the safety, health or rights of the subjects or on the robustness or reliability of the clinical data generated by the investigation, and that the response of no objection will be awaited, in accordance with ISO 14155:2020 and the procedure as laid down in article 75 of MDR 2017/745. All alterations of the present protocol, if accepted, will be communicated to the commission of medical ethics through the use of amendments. All of these amendments will be submitted through the Dycoflow system, and subjected to approval by the commission. The proposed changes will only be implemented if they are approved by the mentioned commission.

10. Deviations from Clinical Investigation Plan

a) Statement specifying that the investigator is not allowed to deviate from the CIP

The investigator shall not deviate from the approved CIP during the study.

b) Procedures for recording, reporting and analysing CIP deviations

Any adjustment to the CIP shall be notified via an amendment as stated in “9 Amendments to the Clinical Investigation Plan”.

c) Notification requirements and time frames

The investigator shall notify and report any deviations from the approved CIP in a timely manner.

d) Corrective and preventive actions and principal investigator disqualification criteria

If the investigator deviates from the CIP without notifying and reporting in a timely manner, the sponsor shall be contacted and disqualification of the investigator is required.

11. Device accountability

a) Description of the procedures for the accountability of investigational devices

The sponsor shall keep records to document the physical location of all investigational devices from shipment of investigational devices to the investigation sites until return or disposal. All of these records are controlled by the principal investigator. The name who received, used, returned or disposed of the device shall be notified, as well as the date of receipt and return of the device.

b) Procedures and particular materials and instructions for the safe return of investigational devices

The investigator, users, rehabilitation doctors and ortho-prosthetists are required to use the investigational device as it is explained in the IFU provided by the manufacturer.
At the end of the clinical investigation, the investigational device will be returned to the manufacturer using its secure case.

12. Statements of compliance

Statement A
We confirm that we are working in accordance with the ethical principles set out in the latest version of the "Helsinki Declaration", the "Good Clinical Practice" and the Belgian law of May 7, 2004 on experiments with human persons.

Statement B
We confirm compliance with the content of the CIP and other regional (e.g. EC) or national regulations.

Statement C
We confirm that the clinical investigation shall not start until approval is obtained from the EC and other regulatory authorities.

Statement D
We confirm that all requirements imposed by the EC or other regulatory authority shall be followed.

Statement E
We confirm that we possess an insurance certificate for Civil liability policy.

Statement F
A clinical investigation agreement is attached to this document.

13. Informed consent process

a) Description of the general process for obtaining informed consent

The approval of subjects to participate in the present investigation will be collected using an informed consent which will be designed using a template of the UZ Brussel.

Participants must go through the 'informed consent' document and approve it by means of a signature before the start of the study. Oral explanation will be given before the start of the study if requested.

b) Description of the informed consent process in circumstances where the subject is unable to give it

Not applicable.

14. Adverse events, adverse device effects, and device deficiencies

a) Definition of adverse events and adverse device effects

There are no disadvantages or risks associated with this research. Moreover, previous experiments with the Talaris Demonstrator (the LUNARIS® prototype, registered on B.U.N. 143201526629) have shown that there is a high willingness and motivation from the participants to participate in the experiments and no side or adverse effects were reported. Risks associated with the investigated medical device are reported in the Risk Management File.

b) Definition of device deficiencies

To date, no device deficiencies have been reported with the LUNARIS® . We are not yet able to give a concise summary of device deficiencies and thus not able to report any deficiency that might have led to a serious adverse event if appropriate action had not been taken, intervention had not occurred, or circumstances had been less fortunate.

c) Definition of serious adverse events

There are no serious adverse events anticipated that will lead to death, injury, permanent impairment or deterioration in health anticipated for this clinical investigation and use of medical device.

d) List of non-reportable adverse events

Not applicable.

e) Time period in which the principal investigator shall report all adverse events and device deficiencies to the sponsor

We ensure that any event as referred to Article 80(2) will be fully recorded by the investigators, manufacturer or other parties, and immediately notified by the sponsor to all competent authorities (including FAMHP) by using the European form in a timely manner.

f) Details of the process for reporting adverse events

We ensure that any event as referred to Article 80(2) will be fully recorded by the investigators, manufacturer or other parties, and immediately notified by the sponsor to all competent authorities (including FAMHP) by using the European form in a timely manner.

g) Details of the process for reporting device deficiencies

We ensure that any event as referred to Article 80(2) will be fully recorded by the investigators, manufacturer or other parties, and immediately notified by the sponsor to all competent authorities (including FAMHP) by using the European form in a timely manner.

h) List of foreseeable adverse events and anticipated adverse device effects

The LUNARIS® is a CE-marked medical device. Therefore, a risk analysis has been performed according to ISO 14971 to ensure that risks associated with the device are identified, evaluated and controlled.

The risk analysis shows that any residual risks which may be associated with the device are acceptable, when weighed against the benefits of the intended use to the lower-limb amputee, and compatible with a high level of protection of health and safety.

i) Emergency contact details for reporting serious adverse events and serious adverse device effects

The principal investigator can be contacted in case of emergency for the reporting of serious adverse events.
The manufacturer can be contacted in case of emergency for the reporting of serious adverse device effects.

j) Information regarding the Data Monitoring Committee

Data will be analysed by the co-investigator(s) and controlled by the principal investigator in assignment of the sponsor. The DMC is the Vrije Universiteit Brussel, Pleinlaan 2, 1050 Brussel.

15. Vulnerable population

Not applicable. Our population are healthy people with a lower limb amputation.

16. Suspension or premature termination of the clinical investigation

a) Criteria and arrangements for suspension or premature termination of the whole clinical investigation or of the clinical investigation in one or more investigation sites

The clinical investigation will be considered as terminated when the proposed sample size is tested and is defined as the last visit of the last subject. As mentioned in the informed consent, subjects can stop or halt their participation at their convenience without any disadvantages.
The end of the clinical investigation or temporary study halt or early termination will be notified to FAMPH. In case of study end reporting this will be done within 15 days (and 24 hours if based on safety grounds). The study report will be submitted within one year of the end of the clinical investigation or within three months if early termination or temporary halt.

b) Criteria for access to and breaking the blinding / masking code in the case of suspension or premature termination of the clinical investigation

The competent authorities shall be notified when the study will be terminated earlier than anticipated. Participation is voluntary, there can be no compulsion in any way. Even after subjects have signed, they can stop their voluntary participation.

c) Requirements for subject follow-up and continued care

There is no follow-up plan considered since subjects perform the experiment with the novel investigated device or with the traditional prosthetic foot used in rehabilitation. Subjects can contact our department during and after the experiment for any further questions.

17. Publication policy

Statement A
The clinical investigation is registered in an open access database: ClinicalTrials.gov.

Statement B
The results of the clinical investigation will be published in an open access database: ClinicalTrials.gov. We are also aiming for a publication in a peer reviewed open access journal.

Statement C
Our publication policy is based on the 'Uniform Requirements for Manuscripts Submitted to Biomedical Journals': authorship credit is based only on (i) substantial contributions to conception and design, or acquisition of data, or analysis and interpretation of data; (ii) drafting the article or revising it critically for important intellectual content; and (iii) final approval of the version to be published. Conditions (i), (ii), and (iii) must all be met. Acquisition of funding, the collection of data, or general supervision of the research group, by themselves, do not justify authorship. Contributors who do not meet the criteria for authorship will be thanked in the acknowledgments section.

Statement D
The submitters of the present protocol proposal declare no financial or other conflicts of interest.
Trial results will be communicated to the general public through the publication of disseminated manuscripts published in scientific journals, and through conference presentations/posters. Authorship of these papers and presentations will only be granted to researchers that performed a substantial contribution to the scientific article. Public access will be granted to the entire protocol through the Clinicaltrials.gov registry. The statistical code and dataset will only be shared upon reasonable request, and the responsible data managers reserve the right to refuse such requests.

18. Bibliography

[1] Lathouwers E, Ampe T, Díaz MA, Meeusen R, De Pauw K. Evaluation of an articulated passive ankle-foot prosthesis. BioMedical Engineering OnLine (2022) 21:28.

[2] Lathouwers E, Díaz MA, Maricot A, Tassignon B, Cherelle C, Cherelle P, Meeusen R, De Pauw K. Therapeutic benefits of lower limb prosthesis: a systematic review. Journal of NeuroEngineering and Rehabilitation (2023) 20:4.

[3] Lathouwers E, Baeyens JP, Tassignon B, Gomez F, Cherelle P, Meeusen R, Vanderborght B, De Pauw K. Continuous relative phases of walking with an articulated passive ankle-foot prosthesis in individuals with a unilateral transfemoral and transtibial amputation: an explorative case-control study. Under review.

[4] Howard CL, Wallace C, Perry B, Stokic DS. Comparison of mobility and user satisfaction between a microprocessor knee and a standard prosthetic knee: a summary of seven single-subject trials. Int J Rehabil Res. 2018;41(1):63-73.

[5] Cox PD, Frengopoulos CA, Hunter SW, Sealy CM, Deathe AB, Payne MWC. Impact of Course Configuration on 6-Minute Walk Test Performance of People with Lower Extremity Amputations. Physiother Can. 2017;69(3):197-203.

[6] Windrich M, Grimmer M, Christ O, Rinderknecht S, Beckerle P. Active lower limb prosthetics: a systematic review of design issues and solutions. BioMedical Engineering OnLine. 2016;15(3):140.

[7] Russell Esposito E, Aldridge Whitehead JM, Wilken JM. Step-to-step transition work during level and inclined walking using passive and powered ankle-foot prostheses. Prosthet Orthot Int. 2016;40(3):311-9.

[8] Voloshina AS, Collins SH. Chapter 23 - Lower limb active prosthetic systems—overview. In: Rosen J, Ferguson PW, editors. Wearable Robotics: Academic Press; 2020. p. 469-86.

[9] Molina-Rueda F, Navarro-Fernández C, Cuesta-Gómez A, Alguacil-Diego IM, Molero-Sánchez A, Carratalá-Tejada M. Neuroplasticity Modifications Following a Lower-Limb Amputation: A Systematic Review. Pm r. 2019;11(12):1326-34.

[10] WMA. Declaration of Helsinki: ethical principles for medical research involving human subjects. J Am Coll Dent. 2014;81(3):14-8.

[11] Boone DA, Coleman KL. Use of the Prosthesis Evaluation Questionnaire (PEQ). JPO: Journal of Prosthetics and Orthotics. 2006;18(6).

[12] Ozer S, Young J, Champ C, Burke M. A systematic review of the diagnostic test accuracy of brief cognitive tests to detect amnestic mild cognitive impairment. Int J Geriatr Psychiatry. 2016;31(11):1139-50.

[13] Rogge A-K, Röder B, Zech A, Hötting K. Exercise-induced neuroplasticity: Balance training increases cortical thickness in visual and vestibular cortical regions. NeuroImage. 2018;179:471-9.

[14] Rogge AK, Röder B, Zech A, Nagel V, Hollander K, Braumann KM, et al. Balance training improves memory and spatial cognition in healthy adults. Sci Rep. 2017;7(1):5661.

[15] Mahon CE, Hendershot BD. Biomechanical accommodation to walking with an ankle–foot prosthesis: An exploratory analysis of novice users with transtibial limb loss within the first year of ambulation. Prosthetics and Orthotics International. 2022:10.1097/PXR.0000000000000124.

[16] Borg GA. Psychophysical bases of perceived exertion. Medicine & science in sports & exercise. 1982.

[17] Lee KA, Hicks G, Nino-Murcia G. Validity and reliability of a scale to assess fatigue. Psychiatry Res. 1991;36(3):291-8.

[18] Hart SG, Staveland LE. Development of NASA-TLX (Task Load Index): Results of Empirical and Theoretical Research. In: Hancock PA, Meshkati N, editors. Advances in Psychology. 52: North-Holland; 1988. p. 139-83.

[19] Agrawal V, Gailey RS, Gaunaurd IA, O'Toole C, Finnieston A, Tolchin R. Comparison of four different categories of prosthetic feet during ramp ambulation in unilateral transtibial amputees. Prosthetics and Orthotics International. 2015;39(5):380-9.

[20] Schmalz T, Blumentritt S, Marx B. Biomechanical analysis of stair ambulation in lower limb amputees. Gait & Posture. 2007;25(2):267-78.

[21] Hunter SW, Frengopoulos C, Holmes J, Viana R, Payne MW. Determining Reliability of a Dual-Task Functional Mobility Protocol for Individuals With Lower Extremity Amputation. Arch Phys Med Rehabil. 2018;99(4):707-12.

[22] Cho YS, Jang SH, Cho JS, Kim MJ, Lee HD, Lee SY, et al. Evaluation of Validity and Reliability of Inertial Measurement Unit-Based Gait Analysis Systems. Ann Rehabil Med. 2018;42(6):872-83.

[23] Kong X-z, Liu Z, Huang L, Wang X, Yang Z, Zhou G, et al. Mapping Individual Brain Networks Using Statistical Similarity in Regional Morphology from MRI. PLOS ONE. 2015;10(11):e0141840.

[24] Tzourio-Mazoyer N, Landeau B, Papathanassiou D, Crivello F, Etard O, Delcroix N, et al. Automated Anatomical Labeling of Activations in SPM Using a Macroscopic Anatomical Parcellation of the MNI MRI Single-Subject Brain. NeuroImage. 2002;15(1):273-89.

[25] Demers L, Monette M, Lapierre Y, Arnold DL, Wolfson C. Reliability, validity, and applicability of the Quebec User Evaluation of Satisfaction with assistive Technology (QUEST 2.0) for adults with multiple sclerosis. Disability and rehabilitation. 2002 Jan 1;24(1-3):21-30.

[26] Demers L, Weiss-Lambrou R, Ska B. The Quebec User Evaluation of Satisfaction with Assistive Technology (QUEST 2.0): An overview and recent progress. Technology and Disability. 2002;14:101-5.

[27] Aledda S, Galeoto G, Fabbrini G, Lucibello L, Tofani M, Conte A, et al. A systematic review of the psychometric properties of Quebec user evaluation of satisfaction with assistive technology (QUEST). Disability and Rehabilitation: Assistive Technology. 2023:1-8.
